# Three year follow-up of an early childhood intervention: is movement skill sustained?

**DOI:** 10.1186/1479-5868-9-127

**Published:** 2012-10-22

**Authors:** Avigdor Zask, Lisa M Barnett, Lauren Rose, Lyndon O Brooks, Maxine Molyneux, Denise Hughes, Jillian Adams, Jo Salmon

**Affiliations:** 1 Health Promotion Unit, Northern New South Wales Local Health District, Lismore, NSW, Australia; 2 School of Health and Social Development, Deakin University, Faculty of Health, Melbourne, VIC, Australia; 3 University Centre for Rural Health North Coast, School of Public Health, University of Sydney, Lismore, NSW, Australia; 4 School of Health and Human Sciences, Southern Cross University, Lismore, NSW, Australia; 5 Marine Ecology Research Centre, Southern Cross University, Lismore, NSW, Australia; 6 Centre for Physical Activity and Nutrition, Deakin University, Faculty of Health, Melbourne, VIC, Australia

**Keywords:** Preschool, Intervention, Object control, Locomotor, Sex

## Abstract

**Background:**

Movement skill competence (e.g. the ability to throw, run and kick) is a potentially important physical activity determinant. However, little is known about the long-term impact of interventions to improve movement skills in early childhood. This study aimed to determine whether intervention preschool children were still more skill proficient than controls three years after a 10 month movement skill focused intervention: ‘Tooty Fruity Vegie in Preschools’.

**Methods:**

Children from 18 intervention and 13 control preschools in NSW, Australia were assessed at ages four (Time1), five (T2) and eight years (T3) for locomotor (run, gallop, hop, leap, horizontal jump, slide) and object control proficiency (strike, bounce, catch, kick, overhand throw, underhand roll) using the Test of Gross Motor Development-2. Multi-level object control and locomotor regression models were fitted with variables time, intervention (yes/no) and a time*intervention interaction. Both models added sex of child and retained if significant, in which case interactions of sex of child with other variables were modelled and retained. SPSS (Version 17.0) was used.

**Results:**

Overall follow-up rate was 29% (163/560). Of the 137 students used in the regression models, 53% were female (n = 73). Intervention girls maintained their object control skill advantage in comparison to controls at T3 (*p* = .002), but intervention boys did not (*p* = .591). At T3, there were no longer intervention/control differences in locomotor skill (*p* = .801).

**Conclusion:**

Early childhood settings should implement movement skill interventions and more intensively target girls and object control skills.

## Background

Increasing children’s physical activity is a worldwide health priority [1]. Movement skill competence, such as jumping, throwing or kicking, is positively associated with physical activity participation in both children and adolescents [2], and is considered a prerequisite to sport participation [3,4]. Preschool age (three to five years old) may be an important time to intervene in movement skill development as this is when movement skills start to develop [4,5]. Studies in this age group have also demonstrated associations between fundamental movement skills and physical activity [6-8], showing that movement skill competence is a potentially important physical activity determinant.

A review of interventions to improve motor skills in children younger than five found that more than half of the 17 studies significantly improved children’s motor skills [9]. A recent meta-analysis [10] also indicates that motor skill interventions are effective compared to control groups. Since these reviews, Tooty Fruity Vegie in Preschools (TFV), a 10-month Australian obesity prevention intervention, found intervention children improved their movement skills significantly more than control children, with a relative improvement of 13% [11]. ‘Munch and Move’ used a similar intervention approach to TFV, and implemented it across the Australian state of New South Wales (NSW). After four months, intervention preschool aged children also had higher movement skills than control children [12].

There is less information about the long term impact of movement skill interventions. Of the intervention review mentioned previously [9] no studies appeared to have a longer term follow-up. The previously mentioned meta-analysis does not report if any studies had a follow-up or retention test [10]. Two studies in young children could be located which implemented a six month retention assessment; one finding that both intervention groups improved significantly in object control skill from baseline to retention in comparison to control children [13], the other finding that intervention children were able to maintain intervention skill benefits [14].

While interventions to improve movement skills in early childhood thus appear to be effective, less is known about their long-term impact (i.e., more than six months post-intervention). To address this gap in the literature, TFV participants were re-assessed three years after the intervention, to determine whether intervention children were still more skill proficient (object control and locomotor) than control children.

## Methods

### Tooty fruity vegie in preschools (TFV)

In 2006/07, children from 18 intervention and 13 control preschools in NSW, Australia participated in TFV; an obesity prevention ten month intervention with a movement skill focus (‘FunMoves’). The ‘FunMoves’ program was games-based and influenced by the ‘Moving with Young Children’ program for preschoolers [15]; but more structured and focusing on the 12 fundamental movement skills in the Test of Gross Motor Development-2 (TGMD-2) [16]. The program consisted of two terms of 10 sessions with each session repeated twice per week. Each session included a warm up and cool down time and a number of short games, usually three. Preschool staff received one day of training and were given a kit with program notes and 30 laminated cards for each of the games to run the program. The cards contained instructions on how to: set up the equipment, play the game, adapt it for different age groups and skill levels, and give verbal cues. In addition, the playground layout and access to sports equipment during free play times were reviewed by project staff and adjustments were made to encourage more physical activity and easier access to equipment. Health professionals held parent workshops on teaching movement skill at home and all preschool parents received written material on ideas for fun games to play with children at home [17].

### Sample selection

The sample for the current study was derived from the 560 intervention and control children for whom we had data from either or both pre and post data collections [11]. The protocol for consent for future follow-up differed for the 2006 (*n* = 220) and 2007 (*n* = 340) children (due to different ethics permissions for each year group). In 2007, parents were asked at the time of intervention consent if they agreed to future follow up and if so, for their telephone details. As a result, 94.4% (321/340) provided consent for future follow-up. In 2006, parents were distributed a consent form for follow up at the end of the intervention via preschools (which needed to be mailed back to study coordinators). As a result 21.4% (47/220) of parents of 2006 children provided consent to future follow-up.

### Data collection

Children were assessed at three time points (four, five and eight years old). They were assessed for movement skill proficiency prior to the TFV intervention in 2006/07 (T1) and again after the intervention (approximately 10 months later) in 2006/07 (T2). Further details on the data collection at these two time points are published elsewhere [11]. Children were assessed for the current study three years later in 2010/11 (T3).

The majority of data (>87%) were collected in the latter half of 2010, with the remainder in the first half of 2011. Ethics approval for the current study was gained from the former North Coast Area Health Service (HREC 487N), the former NSW Department of Education and Training (2010011), Deakin University (2010–154), and the local Catholic Education Office (PT: jw E.2.8.4). Written informed consent was obtained from parents/guardians and assent was gained from children.

### Movement skill measurement

The TGMD-2 is a norm-referenced measure of common gross motor skills, which has been validated for ages three to ten [16]. The TGMD-2 assesses six locomotor skills (running, galloping, hopping, leaping, horizontal jumping, sliding) and six object control skills (striking a stationary ball, stationary dribble, kicking, catching, overhand throwing, and underhand rolling) [16]. The TGMD-2 is a process oriented measure, assessing the components in each skill rather than the outcome or product of the skill execution. The catch and leap have three components, the hop and strike have five, and the remainder of the skills have four components.

The protocol involved children being given a demonstration of the correct technique before assessment. Children were then asked to perform the skill twice. Each attempt was scored with each component receiving a ‘1’ if correctly executed or a ‘0’ if not. The components for the two trials are then summed for each skill and then scores for the six locomotor skills are summed for a composite locomotor score, and scores for the six object control skills are summed for a composite object control score. General encouragement was given but no verbal feedback on skill performance. All children were assessed in the field using live observation by raters trained for that purpose.

Three raters were Health Promotion staff who performed the movement skill testing for TFV in 2006/2007 [17], with an additional rater recruited in 2010. Raters, who were originally trained in 2006, also participated in the repeat 12 h training in 2010 conducted by two expert trainers. In the field, interrater reliability was assessed simultaneously by two raters with no communication between them. Observations were conducted opportunistically (whenever there were two raters available and time permitted). All raters did paired observations with each other. Raters in the field at T3 were rated on their ability to agree on the raw totals for the object control (sum of the six object control skills) and locomotor subsets (sum of the six locomotor skills). An intraclass correlation (ICC) using a one way model for consistency for single measures was chosen to assess interrater agreement. A total of 16% (26/163) of observations were assessed for reliability in the field. For the locomotor raw subtotal, ICC = .73 (CI .49 to .87) and for the object control raw subtotal, ICC = .81 (CI .63 to .91).

### Data management and analysis

The TFV program had a wait list control design which meant that TFV continued after the two cohorts in this paper were assessed. Therefore younger children in control preschools in 2007 who still went to that preschool in 2008 could have received the intervention in 2008 (some children attend preschool for two years). In this state of Australia whilst children cannot attend school until they are at least four years and six months, they do not have to attend by law until they turn six years of age. To minimise this potential source of contamination, control children who could not legally have attended school in 2008 according to NSW Department of Education and Training guidelines (i.e. < five years of age as at 31^st^ July 2008) were excluded from the analysis. Children who were missing complete object control or locomotor subtest data for *both* T1 and T2 were also excluded (this meant that children were included who had T1and T3 OR T2 and T3 data). Missing data occurred because of children being absent on testing days or children not completing the whole locomotor or object control subset.

Each child’s object control and locomotor scores (for T1, T2 and T3) were adjusted to accommodate the age range of children who were tested at each time point and for the different ‘time gaps’ between testing times for each student. This was done by fitting a regression model predicting mean scores by intervention and sex groups separately for each time point and adjusting each child’s score according to his/her age in relation to the child’s age at T1, T2 and T3 respectively, using coefficients from these models.

Object control and locomotor skills scores at T1 and T2, of children who participated at T3, were compared to scores of children who did not to check for selective participation. Two regression models were run with sex, age, intervention (yes/no) / and T3 participation (yes/no) as predictors of locomotor and object control scores. The models were run separately for the T1 and T2 samples so the age adjustment is for that time point. Both models included two way interaction variables.

To examine whether intervention children were still more skill proficient than control children at T3, multi-level regression models were fitted in SPSS to account for clustering [18]. To test intervention effect and adjust for baseline levels, all models included the variables time, intervention (yes/no) and an interaction variable; time*intervention. Sex of child was added to all models and retained if significant, in which case interactions of sex with all other significant variables were modelled and retained if significant.

To ascertain the long term effect the intervention had over and above natural maturation among boys and girls as represented by controls’ scores, a contrast analysis was conducted regarding changes in mean scores over time, by intervention and by sex; i.e. between T1 and T3 mean scores for locomotor and object control scores. Firstly, the change in intervention girls’ object control mean scores between T1 and T3 was compared to the change in control girls’ object control mean scores between T1 and T3, i.e. the difference between the changes was calculated using a contrast test function in the model statement. This was repeated for boys. Finally, the difference between the girls’ and boys’ differences was tested. A Bonferroni adjustment was applied to these three tests’ findings and the level of significance was set to 0.017 (0.05/3). A similar process was repeated for the locomotor skill scores. Predicted values from both the object control and locomotor models were plotted and presented in two figures. SPSS (Version 17.0) was used for analysis.

## Results

### Sample

In 2009/10, parents who had consented to future follow up of their children were contacted regarding their child’s participation in the current study. Of the 321 children from the 2007 cohort, 208 were locatable. Of these, 64.9% (135/208) provided written consent, which meant the follow-up rate of the original cohort was 39.8% (135/340). Of the 2006 children available for follow-up in 2010 (n = 47), 39 were locatable. Of these, 28 parents provided written consent, thus the consent rate for 2006 children was 71.8% (28/39) and the follow-up rate was 12.7% (28/220). In total, 29.1% (163/560) original TFV children were followed up. After excluding control children who may have received an intervention the following year, the number was reduced to 148. A further 11 children who were missing object control and locomotor subtest data for either T1 or T2 were excluded; leaving 137 children.

There was no significant difference in T2 object control scores between children who were followed up at T3 and those who were not (*p* = 0.575). However, at T1, children who were followed up at T3, had significantly higher object control scores than those who were not (1.71 units, *p* = 0.031). There were no significant differences in T1 or T2 locomotor scores between children who were followed up at T3 and those who were not (*p* = 0.077 and *p* = 0.954 for T1 and T2 respectively). Interactions of sex by intervention or intervention by T3 were not significant in either model and were removed.

Of the 137 students, slightly more than half were female (*n* = 73, 53.3%). The mean ages and standard deviations at the three time points were 4.37 years (SD = 0.48) at T1 (*n* = 131), 5.00 years (SD = 0.50) at T2 (*n* = 112) and 8.23 years (SD = 0.65) at T3 (*n*=137). At T3, 54.0% (74/137) were intervention children. Both intervention and control children’s mean locomotor score increased from T1 to T2 (31 to 40) but did not increase much further by T3 (42). In contrast, both intervention and control children’s mean object control score increased by around the same amount at each time point (27 to 34 to 42).

### Object control findings

**Figure 1:**
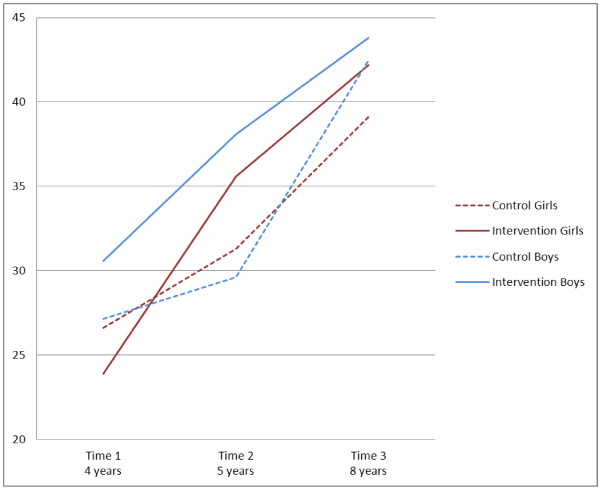
Object control skills - Adjusted mean scores over time for boys and girls.

Table 1 presents parameter estimates for the main variables and their interactions from the final model testing the effect of intervention and sex groupings on adjusted object control scores. It indicates that the changes over time were different for control and intervention students and that difference varied between boys and girls. Predicted age adjusted object control scores from this model are presented in Figure 1 by intervention and sex groups.

**Table 1:** Final models testing effect of intervention and sex groupings on object control and locomotor scores

**Object control model**			
**Variable/Interaction**	**Denominator df**	**F**	**Sig.**
Intercept	181.455	5561.490	<.001
Sex	181.455	7.100	.008
Intervention (Yes/No)	181.455	11.852	.001
Time	183.686	250.781	<.001
Sex * Intervention	181.455	2.695	.102
Sex * Time	183.686	1.226	.296
Intervention *Time	183.686	5.403	.005
Sex * Intervention*Time	183.686	3.622	.029
**Locomotor model**			
Intercept	199.233	8190.919	<.001
Sex	199.272	1.822	.179
Intervention (Yes/No)	199.382	3.482	.063
Time	161.347	73.011	<.001
Sex * Time	161.298	3.990	.020
Intervention *Time	161.376	5.822	.004

Table 2 presents results of the three relevant object control contrast analyses. The notation for contrasts outlines what changes and differences were entered into the model. For example, the first contrast analysed the difference between the changes that occurred among intervention and control girls, so (T3-T1) GI means the change between T1 and T3 among intervention girls. As can be seen in Table 2, the changes in the mean object control skill score between T1 and T3 among intervention girls were significantly larger than the changes among control girls. Intervention and control boys’ changes of mean scores were not significantly different. There was a significant difference between the boys’ and girls’ differences (*p* = 0.014).

**Table 2:** Changes over time estimates for object control and locomotor skills

**Object control**			
**Contrast**	**Estimate**	**SE**	**P-value**
(T3-T1)GI - (T3-T1)GC^1^	5.999	1.945	0.002
(T3-T1)BI - (T3-T1)BC^2^	−1.122	2.083	0.591
[(T3-T1)BI - (T3-T1)BC] - [(T3-T1)GI - (T3-T1)GC] ^3^	−7.121	2.850	0.014
**Locomotor**			
(T3-T1)I - (T3-T1)C^4^	−0.451	1.789	0.801
(T3-T1)B - (T3-T1)G^5^	3.559	1.789	0.049

^1^ The difference between intervention and control girls’ object control changes.

^2^ The difference between intervention and control boys’ object control changes.

^3^ The difference between differences, i.e. the process among boys for object control was different to the one among girls.

^4^ The difference between intervention and control students’ locomotor changes.

^5^ The difference between boys’ and girls’ locomotor changes.

### Locomotor skill findings

**Figure 2:**
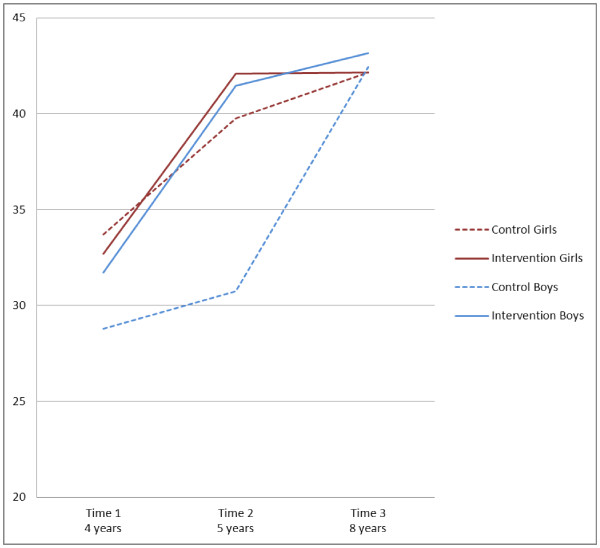
Locomotor skills - Adjusted mean scores over time for boys and girls.

Table 1 also presents parameter estimates for the main variables and their interactions from the final model testing the effect of intervention and sex groupings on adjusted locomotor scores. The pattern of change over time differed between control and intervention students and between boys and girls, but there was no significant interaction between intervention and sex groupings. Predicted age adjusted locomotor scores from this model are presented in Figure 2 by intervention and gender groups.

Table 2 also presents results of the two relevant locomotor skills contrast analyses (since the three-way interaction was not significant, only two contrasts were required). As can be seen, the changes in the mean locomotor skill score between T1 and T3 among intervention students were not significantly different to the changes among controls. There was a 3.6 unit difference between boys’ and girls’ changes, but after a Bonferroni adjustment for two tests, this difference was not significant (*p* = 0.049 which is >*p* = 0.025).

## Discussion

This study followed up children who participated in a preschool intervention to determine whether movement skill improvements gained from the intervention were sustained three years later. It is the only study to our knowledge that has conducted a long term follow up of children who completed a preschool intervention with a movement skill focus. We found at T3 that intervention children had higher scores than control children in terms of their object control skill ability three years after the intervention. However when we looked at the influence of sex, it was clear that intervention girls had maintained their object control skill advantage in comparison to control girls, but that control boys appeared to have ‘caught up’ to the intervention boys. Thus it seems that boys who have not gained object control skills prior to school, may be likely to develop these skills through environmental opportunities provided during early elementary school and/or from home and community life. It has been noted that current Australian physical education (PE) in schools caters to the needs of skilled boys who dominate large-sided team sports in lessons [19] and not for the skill-specific needs and abilities of girls [20].

There is evidence to suggest that many girls do not develop object control skills. In a study of children in early elementary school, boys performed better than girls in all of the assessed object control skills (catch, kick, throw and strike) [21]. This study suggested that these differences were likely to be environmental and that if girls were provided with the same opportunities for instruction, feedback, practice and encouragement, that the differences in skill proficiency between girls and boys could be reduced [21]. A study in Aboriginal Australian children lends support to the importance of environment in throwing ability. This study found the differences in throwing mechanics and velocity were smaller between girls and boys than that found in other cultural groups, which the authors suggest may be due to different cultural throwing expectations before European settlement [22]. Our study also lends weight to the importance of environment in skill ability, as the girls who received the preschool TFV intervention did manage to keep ahead of the control girls in terms of their object control skills. The Physical Activity and Skills study (PASS); a follow-up study of children in late elementary school, found that by late adolescence, only one in two girls could perform the overhand throw and only one out of four could perform the kick [23]. Girls started much less competent as a group than the boys in the kick, and fewer girls than boys improved over the six year time period [23]. Even though similar proportions of girls improved over time performing the overhand throw, they did not ‘catch up’ to the boys, because they were less proficient in childhood [23]. In fact, only 13% of girls had reached mastery or near mastery in the overhand throw at age 10 [23]. Instruction and adequate opportunity for practice are significant factors in the development of throwing techniques in children [24], so this indicates girls may not be receiving enough practice time in these sorts of skills [25-27].

The PASS suggested that skill developed (for example in throwing) prior to age 10 may be of great importance to subsequent movement skill competency [23], and that low-skilled girls may need intervention whilst in early elementary school. The current study also supports this suggestion, as the preschool TFV intervention does appear to have helped the young girls in this study gain these skills at what may be an opportune time for skill development.

Of the limited follow-up studies of movement skill interventions, there is some evidence to support that girls can benefit more than boys. Switch Play followed up children 12 months after an elementary school intervention and found that intervention girls recorded significantly higher motor skill z-scores [28]. Switch Play assessed five skills, three object control (overhand throw, strike, kick) and two locomotor (run, vertical jump). However Switch Play assessed movement skill as a total quotient, rather than in terms of object control and locomotor skill subsets, so it is not known whether the skill improvement in girls can be attributed to certain skill types.

The current study found there were no longer any differences between intervention and control children in locomotor skill ability three years after the TFV intervention. In the years following the intervention, control boys and girls both ‘caught up’ to intervention children in locomotor skill ability. This finding indicates that whilst locomotor skill ability can be increased in an intervention setting, that these skills may be subsequently gained through the environmental influences children are exposed to in early elementary school and in their home and community, e.g. school physical education, opportunities to exercise and enrolment in out of school activities and sports. Interestingly, it may be that the school physical education and environmental opportunities already provide what is required for both girls and boys to develop locomotor skill; as opposed to object control skill. Also there may be a ceiling effect operating as children in this study had more room to improve in object control skills than in locomotor skills.

It has been shown there is little relationship between locomotor proficiency in childhood and in adolescence, whereas childhood object control proficiency does help to explain subsequent object control skill [23]. This may indicate that locomotor ability is more variable than object control ability and possibly more influenced by other factors such as weight [29]. This also illustrates that gaining object control skill in childhood is perhaps more important than gaining locomotor skill, as object control skill tracks through to adolescence.

If movement skill competence translates to subsequent physical activity, then there is a case for interventions to improve movement skill competency in typically developing children. Switch Play demonstrated that children in the intervention groups had significant positive average differences in physical activity, compared to controls, with the greatest effects for those in the movement skill group [28]. While the PASS found object control proficiency predicted subsequent physical activity using the whole sample [30], it did not find this effect for intervention children [31]. Interestingly, Switch Play, like the PASS, followed up 10 year old children, which reinforces the notion that possessing skills by this age is crucial in terms of impacting upon subsequent physical activity behaviour. Further longitudinal research could investigate the benefits of increasing children’s movement skill through interventions in terms of subsequent perceived sports competence and subsequent physical activity.

The strengths of this study are its longitudinal design, a good-sized cohort of boys and girls initially assessed at four years of age, and the use of instruments to assess locomotor and object control that have been previously validated in the literature. The main limitation was the follow up rate which was under a third (29%) of original participants. The low follow up rate can be attributed to two factors. One, the study was not conceived as a longitudinal study and 2) due to the differing ethics procedure involved for each year group, many potential children were lost from the study (i.e. 79% of the 2006 parents did not give permission for follow-up of children compared to 6% of 2007 parents). Also the length of the follow-up period meant many children may have moved out of the region. Nevertheless, there does not appear to be meaningful skill differences between those children followed up and those not. There were no significant differences in T1 or T2 locomotor scores between children who were followed up at T3 and those who were not. At T1, children who were followed up at T3 had higher object control scores than those who were not followed up. However, at T2, there were no significant differences in object control scores between children who were followed up at T3 and those who were not. A minor limitation is that the interrater reliability for the locomotor skills was a little lower than that reported in similar field based assessment studies [32,33].

## Conclusions

Our results have direct implications for policies regarding early childhood movement programs. A key implication of our study is that early childhood settings should target movement skills and that girls should be targeted more intensively. The children who were targeted in this intervention can be considered ‘average’ children. The T1 gross motor mean TGMD-2 quotient scores for boys and girls and for intervention and control children were all within the ‘average’ TGMD-2 range (i.e. 90–110) [11]. Thus, ‘average’ preschool aged girls can be targeted to improve their object control skills and this improvement can be retained through to the middle of elementary school. A further recommendation is that object control skills be more specifically targeted than locomotor skills, as it seems children in this study gained these skills anyway. However it must be noted that the acceleration of locomotor skills may have helped children in other ways that this study did not assess, for instance to have higher perceived sports competence. Therefore it may be more appropriate to integrate locomotor and object control skills into learning activities as a way to promote object control skills, but not ignore locomotor skills.

Currently in Australia, the national physical activity recommendations for preschoolers are to provide children with opportunities to practice development of locomotor, stability and object control skills and to give feedback and encouragement [34]. Yet, the Australian Government Healthy Eating and Physical Activity Guidelines for Early Childhood Settings, whilst recommending preschoolers should be physically active every day for at least three hours, do not specify movement skill development [34]. The Australian national quality standards for early childhood education also do not stipulate movement skill development [35]. If movement skill education became part of standard early childhood delivery this may have the potential to greatly impact on girls’ object control skill development.

## Competing interests

Funding was received from New South Wales Ministry of Health for this study. There is no conflict of interest for any of the eight authors. There are no conflicts of interest to declare.

## Authors’ contributions

AZ conceived of study design, contributed to data collection, data analysis and results interpretation, and drafted the manuscript; LMB conceived of study design, contributed to data analysis and results interpretation, and drafted the manuscript; LR contributed to data collection, data analysis and results interpretation, and commented on manuscript drafts; LOB conducted data analysis and contributed to results interpretation, and commented on manuscript drafts; MM managed and contributed to data collection and commented on manuscript drafts, DH contributed to data collection and data management and commented on manuscript drafts, JA contributed to study design and commented on manuscript drafts, JS contributed to study design, contributed to results interpretation, and commented on manuscript drafts. All authors read and approved the final manuscript.
